# Low CD1c + myeloid dendritic cell counts correlated with a high risk of rapid disease progression during early HIV-1 infection

**DOI:** 10.1186/s12879-015-1092-8

**Published:** 2015-08-19

**Authors:** Yingying Diao, Wenqing Geng, Xuejie Fan, Hualu Cui, Hong Sun, Yongjun Jiang, Yanan Wang, Amy Sun, Hong Shang

**Affiliations:** Department of Laboratory Medicine, The First Affiliated Hospital, China Medical University, Shenyang, Liaoning Province China; Department of Laboratory Medicine, Key Laboratory of AIDS Immunology of National Health and Family Planning Commission, The First Affiliated Hospital, China Medical University, Shenyang, Liaoning Province China; Collaborative Innovation Center for Diagnosis and Treatment of Infectious Diseases, Hangzhou, Zhejiang Province China

## Abstract

**Background:**

During early HIV-1 infection (EHI), the interaction between the immune response and the virus determines disease progression. Although CD1c + myeloid dendritic cells (mDCs) can trigger the immune response, the relationship between CD1c + mDC alteration and disease progression has not yet been defined.

**Methods:**

EHI changes in CD1c + mDC counts, surface marker (CD40, CD86, CD83) expression, and IL-12 secretion were assessed by flow cytometry in 29 patients.

**Results:**

When compared with the normal controls, patients with EHI displayed significantly lower CD1c + mDC counts and IL-12 secretion and increased surface markers. CD1c + mDC counts were positively correlated with CD4+ T cell counts and inversely associated with viral loads. IL-12 secretion was only positively associated with CD4+ T cell counts. Rapid progressors had lower counts, CD86 expression, and IL-12 secretion of CD1c + mDCs comparing with typical progressors. Kaplan-Meier analysis and Cox regression models suggested patients with low CD1c + mDC counts (<10 cells/μL) had a 4-fold higher risk of rapid disease progression than those with high CD1c + mDC counts. However, no relationship was found between surface markers or IL-12 secretion and disease progression.

**Conclusions:**

During EHI, patients with low CD1c + mDC counts were more likely to experience rapid disease progression than those with high CD1c + mDC counts.

## Background

Myeloid dendritic cells (mDCs) are a subset of dendritic cells (DCs) responsible for presenting antigens. They play critical roles in the induction of innate and acquired immune responses to viruses [1–4]. mDCs consist of heterogeneous cell populations, including the main subpopulation CD1c + mDCs [5, 6]. CD1c + mDCs are defined as lineage-negative (including CD3, CD14, CD16, CD19, CD20, and CD56), HLA-DR-positive, and CD1c + CD11c + cells. As one subset of mDCs, CD1c + mDCs can be stimulated by various pathogens (such as HIV-1), which results in the increase of major histocompatibility complex (MHC) molecules and surface markers (CD40, CD86, CD83) [7, 8], and secretion of large amounts of IL-12. Such changes direct Th1 cell development and potently prime cytotoxic lymphocyte (CTLs) [3]. If CD1c + mDC stimulation is suppressed, HIV-1 infection will progress to AIDS rapidly [9].

Evaluation of HIV-1 disease progression is important to identify early risk factors of disease progression and improve clinical management. HIV-1 typical progressors (TPs) take 8 to 10 years generally to progress to AIDS without antiretroviral therapy [10]. Rapid progressors (RPs), however, progress to AIDS quicker or meet the criteria for initiation of antiretroviral treatment within the first year after seroconversion [10, 11]. The pathogenesis of HIV-infected patients with rapid progression remains unclear. Early HIV-1 infection (EHI) occurs within the first few months of HIV-1 infection typically; during EHI, HIV-specific immune responses particularly CD8+ T cells affect disease progression [12–17]. CD1c + mDCs initiate and modulate immune responses [18], which can induce HIV-1-specific CD8+ T cells to produce type I interferons and slow down disease progression in HIV-1 elite controllers [19]. Previous studies have shown that CD1c + mDCs were reduced markedly in number during EHI, and HIV-exposed CD1c + mDCs were not fully activated and showed defective IL-12 production [20]. The relationship between CD1c + mDC alteration in EHI and HIV-1 disease progression is unknown. However, the loss of mDCs in early simian immunodeficiency virus (SIV) infection seems to accelerate disease progression [21]. Therefore, we decided to focus on the relationship between CD1c + mDC alteration during EHI and disease progression.

In this study, we sought to determine whether CD1c + mDC alteration was consistent with CD4+ T cell counts or viral loads, whether CD1c + mDC alteration was evident in RPs compared with TPs, and whether CD1c + mDC alteration was associated with rapid disease progression.

## Methods

### Subjects

Twenty-nine HIV-1 infected patients with EHI from a cohort of men who have sex with men (MSMs) were recruited for this study [10, 22]. All patients met the criteria for “early HIV-1 infection” (Fiebig stage V-VI [23]). Beginning in May 2009, all subjects enrolled had their viral loads and CD4+ T cell counts measured at study entry (baseline), at week 1, 2, 3, 7, 11, and 23, and once every 3 months follow-ups starting from week 23, and trial endpoint is January 2013. CD1c+ mDC counts measurement was performed at baseline. RPs were defined as HIV-1 infected individuals who had CD4+ T cell counts <350 cells/μL within the first year of infection [10, 11]. TPs were subjects whose CD4+ T cell counts remained ≥500 cells/μL at the 12-month follow-up visit [10]. Eleven subjects consented to collect large numbers of peripheral blood mononuclear cells (PBMCs) for a more extensive analysis on expression of surface markers and IL-12 secretion by CD1c + mDCs. 11 normal controls (NCs) were age-matched, healthy, HIV-1 negative individuals. None of the study subjects had syphilis or was infected with hepatitis B or C virus. The study protocol and informed consent forms were approved by Ethical Review Board of the First Affiliated Hospital of China Medical University. Informed consents were obtained from all study subjects.

### Counting CD1c + mDCs

CD1c + mDC counts were monitored with a single-platform TruCOUNT assay [24]. A total of 100 μL anticoagulated whole blood was incubated with the following antibodies for 15 min in the dark at room temperature: lineage (CD3/CD14/CD16/CD19/CD20/ CD56)-FITC (all antibodies from BD Bioscience, San Jose, CA, unless otherwise noted), CD45-PerCP, HLA-DR-APC/CY7, and CD1c-PE (Biolegend, San Diego, CA). Each tube receiving 450 μl of FACS Lysing Solution was vortexed gently, and incubated for another 15 min in the dark at room temperature. At least 200,000 events were collected from each sample with BD FACS Calibur, and the data obtained were analyzed with CellQuest.

### Expression of surface markers

Frozen PBMCs were thawed rapidly, washed, and then exposed to antibodies against lineage markers CD3-PerCP/CY5.5, CD19-PerCP/CY5.5, CD14-PerCP/CY5.5 (Biolegend, San Diego, CA), CD16-PerCP/CY5.5 (Biolegend, San Diego, CA), CD56-PerCP/CY5.5 (Biolegend, San Diego, CA), HLA-DR-APC/CY7, CD11c-PE/CY7 (eBioscience, San Diego, CA), CD1c-PE (Biolegend, San Diego, CA), CD40-FITC, CD86-APC, and CD83-APC. PBMCs were incubated for 30 min in the dark at 4 °C. Then, the samples were washed and fixed with 1 % paraformaldehyde. At least 200,000 events were collected from each sample with BD FACS LSRII (BD Biosciences, San Diego, CA), and the data obtained were analyzed with FlowJo (Treestar, Ashland, OR).

#### Cytokine IL-12 Secretion Assays

Frozen PBMCs were thawed rapidly, washed, and then stimulated with 10 μM R848 (Invitrogen, Carlsbad, CA) for 22 h [25]. Brefeldin A was added 10 h prior to the end of the incubation period. PBMCs were exposed to antibodies against lineage markers CD3-PerCP/CY5.5, CD19-PerCP/CY5.5, CD14-PerCP/CY5.5 (Biolegend, San Diego, CA), CD16-PerCP/CY5.5 (Biolegend, San Diego, CA), CD56-PerCP/CY5.5 (Biolegend, San Diego, CA), HLA-DR-APC/CY7, CD11c-PE/CY7 (eBioscience, San Diego, CA), CD1c-PE (Biolegend, San Diego, CA) for 30 min in the dark at 4 °C. Each tube receiving 250 μL of Cytofix/Cytoperm solution was vortexed gently, and incubated for 20 min in the dark at 4 °C. The samples were then washed, exposed to antibodies against IL-12, incubated for 30 min in the dark at 4 °C, and fixed with 1 % paraformaldehyde. At least 200,000 events were collected from each sample with BD FACS LSRII, and the data obtained were analyzed with FlowJo.

### Statistical analysis

Questionnaires were entered twice, and then checked for accuracy with EpiData software (the EpiData Association, Odense, Denmark, version 3.02). Data were analyzed with SPSS 17.0 software. Categorical data were described, and analyzed by frequency and a chi-square test. An independent *t* test was used to analyze normally distributed continuous variables between the two groups. The Mann–Whitney *U* test was used for analyzing non-normally distributed continuous variables. P values less than 0.05 were considered as statistically significant. For the association between baseline covariates and the primary end point (CD4+ T cell count < 350 cells/μL or the commencement of ART), Kaplan-Meier plots and Cox regression models were constructed, and subsequently adjusted for baseline covariates.

## Results

### The baseline characteristics of HIV-1 infected subjects with EHI

Baseline characteristics of 29 EHI individuals were described briefly in Table 1. Eighteen subjects (CD4+ T cell counts <350 cells/μL) were classified as rapid progressors (RPs), whereas 11 subjects (CD4+ T cell counts remained ≥500 cells/μL) belonged to typical progressors (TPs). In this study, none of the subjects has received ART until after the end of the follow-up. CD4+ T cell counts of RPs decreased rapidly at a rate of 46 cells/μL/year, before patients (RPs) received ART after infection for 2 years. By the time patients (RPs) received ART, CD4+ T cell counts had already decreased to a median of 92 cells/μL by comparing with those during baseline (*p* = 0.007). Viral loads of RPs increased at a rate of 0.219 log_10_ copies/mL/year over 2 years after infection. When patients (RPs) received ART, viral loads slightly increased by comparing with those during baseline. However, CD4+ T cell counts in TPs decreased slowly at a rate of 33 cells/μL/year, before patients (TPs) received ART after 3 years of infection. When patients (TPs) received ART, CD4+ T cell counts decreased to a median of 99 cells/μL by comparing with those during baseline (*p* = 0.007). Viral loads of TPs increased at a rate of 0.110 log_10_ copies/mL/year over 3 years after infection. When patients (TPs) received ART, viral loads slightly increased by comparing with those during baseline. There were no obvious changes in CD4+ T cell percentages over time in RPs or TPs. After RPs received ART for approximately 1 year, viral loads decreased significantly (*p* = 0.019), but there were no significant changes in CD4+ T cell counts or CD4+ T cell percentages. There were no obvious changes in TPs; however, the ART observation time was very short.

**Table 1:** Clinical and epidemiological characteristics of seronegative individuals, EHI patients, RPs, and TPs

	NCs *N* = 11	EHI *N* = 29	RPs *N* = 18	TPs *N* = 11	P (P^a^/ P^b^)
Age, years	31 (28–35)	28 (22–39)	37 (23–42)	25 (22–35)	NS/NS
Han ethnicity, no., (%)	11 (100 %)	29 (100 %)	18 (100 %)	11 (100 %)	NS/NS
Male gender, no., (%)	11 (100 %)	29 (100 %)	18 (100 %)	11 (100 %)	NS/NS
Follow-up days, days	NA	1149 (862–1297)	1091 (821–1311)	1171 (990–1284)	NA/NS
At baseline					
Estimated time at baseline, days	NA	55 (36–100)	69 (35–102)	53 (37–81)	NA/NS
Baseline CD4 + T cell counts, cells/μL	728 (568–876)	490 (312–568)	337 (265–520)	586 (499–685)	0.015/0.000
Baseline HIV-1 RNA load, log_10_ copies/mL	NA	4.70 (3.78–5.50)	5.02 (4.24–5.78)	4.03 (3.06–5.15)	NA/NS
At 12-month follow-up visit					
CD4 + T cell counts, cells/μL	NA	329 (271–539)	298 (239–328)	599 (525–732)	NA/0.000
HIV-1 RNA load, log_10_ copies/mL	NA	4.50 (3.64–4.85)	4.55 (3.19–4.85)	4.45 (3.73–4.91)	NA/0.902
At primary endpoint					
Estimated time at primary endpoint, days	NA	340 (104–1149)	123 (79–272)	1171 (990–1284)	NA/0.000
CD4 + T cell counts, cells/μL	NA	321 (281–467)	311 (274–342)	422 (309–541)	NA/0.027
HIV-1 RNA load, log_10_ copies/mL	NA	4.59 (3.89–5.27)	4.59 (3.79–4.71)	4.49 (3.88–5.37)	NA/0.152

Data are values (% of nonmissing values) for categorical variables and medians (interquartile range) for continuous variables

*NCs* normal controls, *EHI* subjects with early HIV-1 infection, *RPs* rapid progressors, *TPs* typical progressors, *NA* not applicable, *NS* not significant

*P*-value from independent *t* test for normally distributed continuous variables or Mann–Whitney *U* test for non-normally distributed continuous variables. P^a^ comparisons between NCs and EHI, P^b^ comparisons between RPs and TPs

The main HIV-1 subtype among the individuals was HIV_AE. The patients had a median viral load of 4.70 (3.78–5.50) log_10_ copies/mL, and an median CD4+ T cell count of 490 (312–568) cells/μL. CD4+ T cell counts in EHI subjects were significantly lower than those in NCs (*p* = 0.015) (Table 1).

The data of baseline demographic characteristics (age, race, gender) and the follow-up durations were comparable between RPs and TPs. At baseline, CD4+ T cell counts in RPs were significantly lower than those in TPs (*p* < 0.001), but viral load levels were similar (Table 1).

### CD1c + mDC counts and IL-12 secretion in EHI subjects were associated with markers of disease progression

**Fig. 1:**
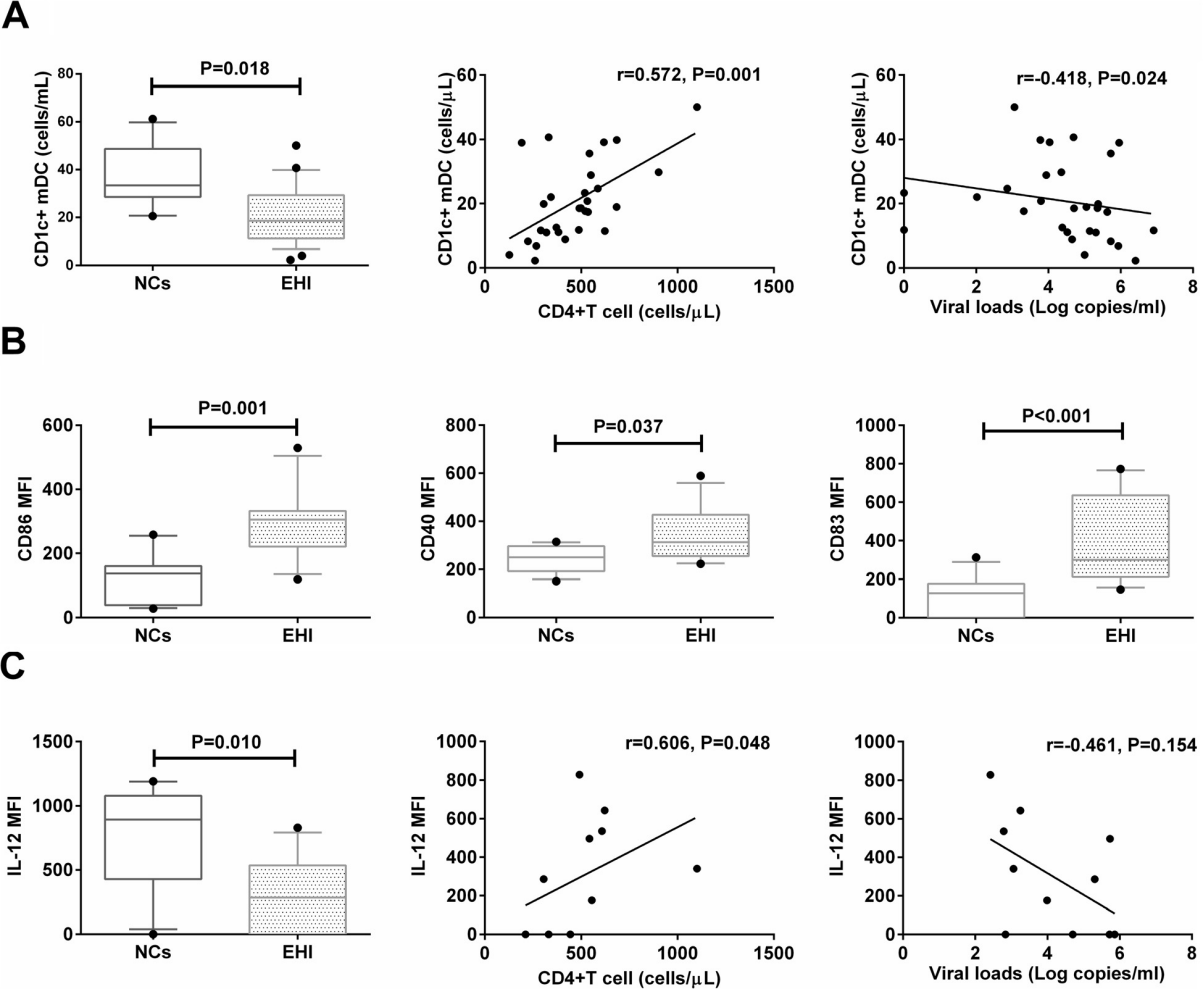
The correlation between CD1c + mDC counts or IL-12 and CD4+ T cell counts or viral loads. **a** CD1c + mDC counts in individuals with EHI and in NCs, and correlations between CD1c + mDC counts and corresponding CD4+ T cell counts or viral loads. **b** Coreceptor (CD86, CD40 and CD83) expression of CD1c + mDC in individuals with EHI and in NCs. **c** IL-12 secretion in individuals with EHI and in NCs, and associations between intracellular IL-12 secretion and corresponding CD4 + T cell counts or viral loads

To assess whether changes in CD1c + mDC counts, surface marker expression, and IL-12 secretion would be evident during EHI, we found that patients with EHI had significantly lower CD1c + mDC counts and IL-12 secretion levels compared with NCs (*p* = 0.018, *p* = 0.010) (Fig. 1a and c). CD1c + mDC had significantly higher expression of CD86, CD40, and CD83 during EHI compared with CD1c + mDC of NCs (*p* = 0.001, *p* = 0.037, and *p* < 0.001, respectively) (Fig. 1b).

Next, we evaluated whether viral loads and CD4+ T cell counts, the only validated markers of progression used in HIV-1 clinic [26, 27], were correlated with CD1c + mDC counts or IL-12 secretion. CD1c + mDC counts were positively associated with CD4+ T cell counts (*r* = 0.572, *p* = 0.001), and inversely associated with viral loads (*r* = −0.418, *p* = 0.024) (Fig. 1a). However, IL-12 secretion was only positively associated with CD4+ T cell counts (*r* = 0.606, *p* = 0.048), but not with viral loads (*r* = −0.461, *p* = 0.154) (Fig. 1c). There was no relationship between surface molecule expression and CD4+ T cell counts or viral loads (data not shown).

### CD1c + mDC counts and IL-12 secretion level were lower in RPs

**Fig. 2:**
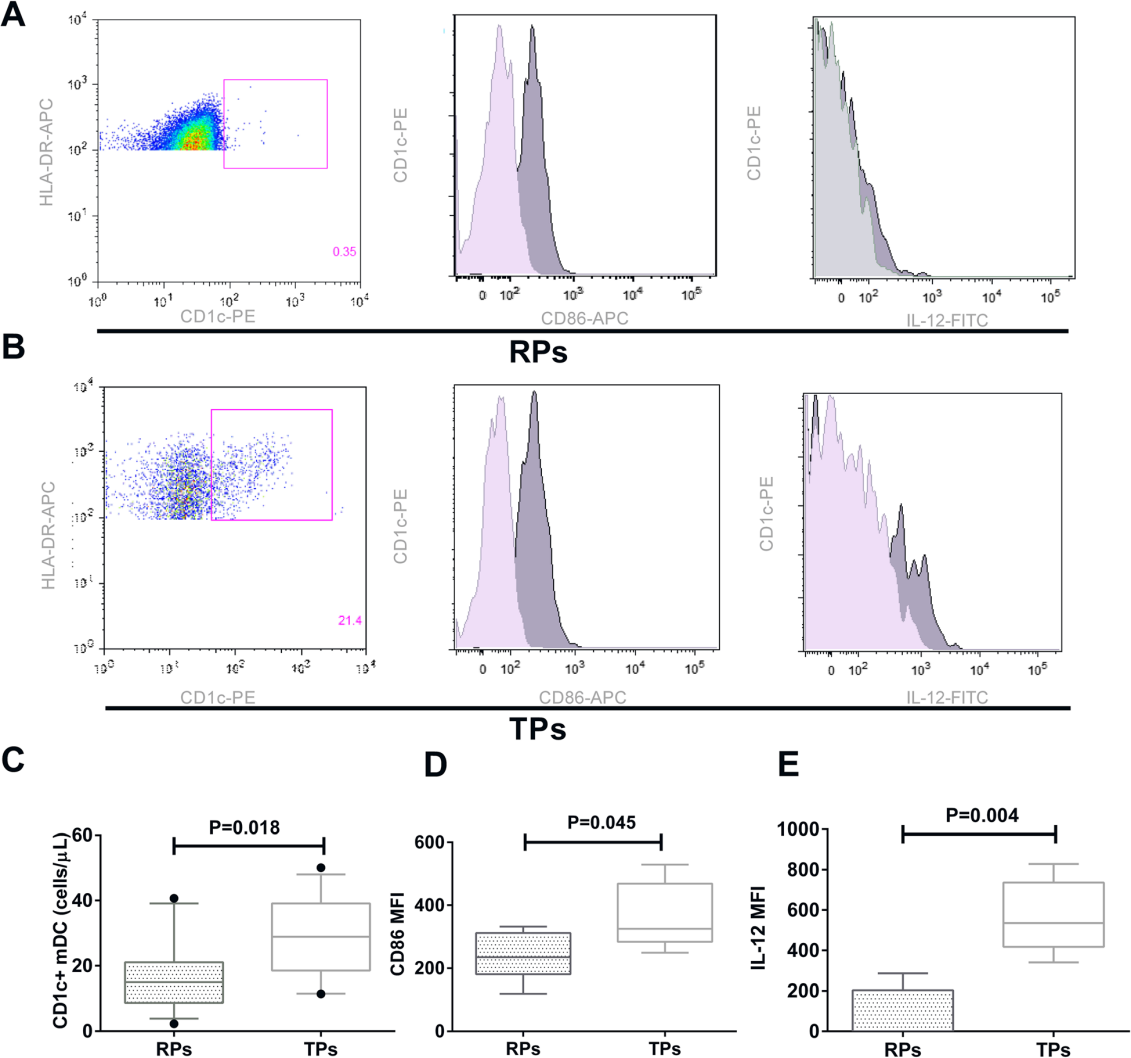
Abnormally low CD1c + mDC counts, CD86 expression, and IL-12 secretion in RPs compared with TPs. **a** Flow cytometry plots of CD1c + mDC counts, CD86 expression, and IL-12 secretion in RPs. Light gray histograms represented isotype control staining for CD86 expression or IL-12 secretion. **b** Flow cytometry plots of CD1c + mDC counts, CD86 expression, and IL-12 secretion in TPs. Light gray histograms represented isotype control staining for CD86 expression or IL-12 secretion. **c** CD1c + mDC counts in RPs and in TPs. **d** Expression of CD86 on CD1c + mDC in RPs compared with TPs. **e** Intracellular secretion of IL-12 in CD1c + mDCs from RPs and TPs after stimulation with R848

Since the disease in RPs progressed more rapidly, we next determined whether abnormally low CD1c + mDC counts and expression of CD40, CD86, and CD83, and IL-12 secretion would be evident in RPs. CD1c + mDC counts in RPs were significantly lower than those in TPs (*p* = 0.018) (Fig. 2a, 2b, 2c). In addition to CD40 and CD83, RPs also had lower CD86 expression than TPs (*p* = 0.045) (Fig. 2a, 2b, 2d). CD1c + mDC secretion of IL-12 in RPs was also significantly lower than TPs stimulated with R848 (*p* = 0.004) (Fig. 2a, 2b, 2e).

### Association between CD1c + mDC counts in EHI subjects and disease progression

**Fig. 3:**
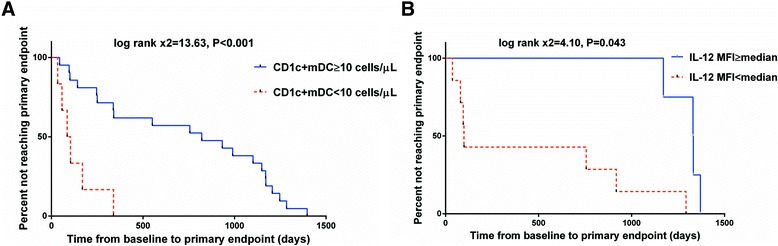
Time from baseline to CD4+ T cell count of less than 350 cells/μL or commencement of ART. **a** Kaplan-Meier survival plots for survival time of low and high CD1c + mDC count groups. **b** Kaplan-Meier survival plots for survival time of low and high IL-12 secretion groups

Disease progression was defined according to the primary endpoint of the CD4+ T cell count of 350 cells/μL or the commencement of ART. All patients received ART at the trail endpoint. We carried out Kaplan-Meier survival analyses, and stratified according to CD1c + mDC counts (<10 cells/μL or ≥10 cells/μL) or median IL-12 level at baseline. There was a significant acceleration in clinical progression in those with lower CD1c + mDC counts (log rank × 2 = 13.63, *p* < 0.001) (Fig. 3a) or IL-12 level (log rank × 2 = 4.10, *p* = 0.043) (Fig. 3b). The median time from baseline to primary endpoint for low and high CD1c + mDC counts was 95.0 (IQR 52.0–211.5) and 821.0 (IQR 248.5-1,170.5) days, respectively; for low and high IL-12 levels, it was 77.0 (IQR 36.0–183.8) and 1,101.0 (IQR 400.0–1481.0) days, respectively (Fig. 3).

Univariable cox analyses showed low CD1c + mDC counts [*HR* = 6.52 (2.11–20.14); *p* = 0.001], low IL-12 level [*HR* = 8.83 (1.01–77.29); *p* = 0.049], and low CD4+ T cell counts (≤350 cells/μL) [*HR* = 0.34 (0.14–0.82); *p* = 0.016], which predicted time to primary endpoint (Table 2). Multivariable analyses were carried out with the baseline covariates, CD1c + mDC counts, viral loads, and CD4+ T cell counts. Low CD1c + mDC counts [4.76 (1.40–16.18); *p* = 0.012] but not low CD4+ counts [*HR* = 0.39 (0.11–1.37); *p* = 0.140] or high viral loads (>5 log_10_ copies/mL) [*HR* = 0.55 (0.14–2.10); *p* = 0.383] predicted time to primary endpoint (Table 2). In a parallel multivariable analysis, low IL-12 level was not significantly associated with the trial endpoint.

**Table 2:** Association between primary endpoint (CD4+ T cell count <350 cells/ml or commencement of long-term ART) and baseline characteristics

	Univariable unadjusted		Multivariable adjusted^a^	
Covariate	HR (95 % CI)	P^a^	HR (95 % CI)	P^b^
CD1c + mDC count (<10 versus ≥10 cells/μL)	6.52 (2.11–20.14)	0.001	4.76 (1.40–16.18)	0.012
CD4+ T cell count (<350 versus ≥350 cells/μL)	0.34 (0.14–0.82)	0.016	0.39 (0.11–1.37)	0.140
Viral load (<5 versus ≥5 log10 copies/mL)	1.50 (0.67–3.36)	0.320	0.55 (0.14–2.10)	0.383

*CI* confidence interval, *HR* hazard ratio. *P*-value from Wald test

*P* ^*a*^-value is from univariable cox analyses. *P* ^*b*^-value is from multivariable cox analyses

^a^Results adjusted for CD1c + mDC counts, CD4+ T cell counts, and HIV viral loads

## Discussion

Early plasmacytoid dendritic cell changes predict plasma HIV load rebound during primary infection [28], but the specific role of mDCs during early HIV-1 infection remains poorly defined. Previous data from macaques revealed that the loss of mDCs during early SIV infection was predictive of disease progression. Therefore, the goal of this study was to explore the correlation between CD1c + mDC alteration in patients with EHI and disease progression. We found CD1c + mDC counts and IL-12 secretion were positively associated with CD4+ T cell counts, and CD1c + mDC counts were inversely associated with viral loads. Furthermore, CD1c + mDC counts and IL-12 secretion level in RPs decreased comparing with those in TPs. Finally, low CD1c + mDC counts (<10 cells/μL) were associated with an increased risk of more rapid disease progression, suggesting a possible target for intervention.

While CD1c + mDC counts and IL-12 secretion decreased, the expression of several surface molecules (CD40, CD86, CD83) increased during EHI. The results were consistent with the previous studies that have demonstrated IL-12 secretion defects in mDCs, CD86 and CD83 up-regulation on monocyte-derived dendritic cells (MDDCs) [9, 10, 29–33]. However, in contrast to our results, a previous study found that mDCs of patients with EHI produced significantly higher levels of IL-12 than normal controls [34]. It might be due to the presence or absence of the circulating immunomodulatory environment within the *in vitro* culture system [34]. The influence of the extracellular milieu or apoptotic microparticles, derived from dying cells during acute HIV-1 infection, has been shown to suppress mDC function [28, 29, 35].

To our knowledge, no previous study has found that changes in mDCs correlated with either CD4+ T cell counts or viral loads during EHI [10, 31]. Our results revealed that it might be due to the heterogeneity of mDCs. CD1c + mDCs, one dominant subset of mDCs, were most closely related to CD4+ T cell counts and HIV-1 viral loads during EHI. Patients with greater CD1c + mDC counts and IL-12 secretion had higher CD4+ T cell counts and patients with greater CD1c + mDC counts had lower viral loads. However, there was no correlation between the expression of several surface markers (CD40, CD86, CD83) and CD4+ T cell counts or viral loads.

Our results also showed that CD1c + mDC counts, CD86 expression, and IL-12 secretion were significantly lower in RPs during EHI. Other studies have found similar results for mDCs and CD11c + CD16- mDCs [10, 36]. Thus, increased CD1c + mDC counts, CD86 expression, and IL-12 secretion seemed to be associated with slower disease progression. The decrease in CD1c + mDC counts observed in HIV-1-infected individuals could be due to HIV-1-induced cell death or recruitment to lymphoid organs [36]. While the underlying cause of the faster decline of CD1c + mDC counts in RPs was unclear, the virus might induce more cell death or recruit more CD1c + mDCs to lymphoid organs. However, less IL-12 secretion in RPs could be due to a lower CD1c + mDC count or a dysfunction induced by HIV-1. Interestingly, we found that CD86 expression was higher in patients with EHI than that in normal controls, which implied that CD1c + mDCs were activated by the virus. However, CD86 expression on CD1c + mDCs was reduced in RPs by comparing with that in TPs, indicated that the virus might inactivate CD1c + mDCs. Although the mechanism was unknown, the decline of CD86 expression has been shown to inhibit CD1c + mDC maturation and suppress immune response [36].

Low CD1c + mDC counts (<10 cells/μL) were associated with an increased risk of more rapid disease progression. The patients with low CD1c + mDC counts had 4-fold higher odds of rapid disease progression than those with high CD1c + mDC counts. It could be due to several different mechanisms. Firstly, CD1c + mDCs had cytotoxic properties [37], so the decrease in CD1c + mDCs might compromise the ability of the immune system to kill virus-infected cells. Secondly, HIV-1 infected CD1c + mDCs selectively in human blood. The infected CD1c + mDCs mobilized from the blood to the lymph nodes, and then underwent apoptosis [21]. The decline of CD1c + mDCs would weaken the immune response. Thirdly, downregulation of CD1c molecules by HIV-1 infection weakened the ability of CD1c-restricted T cells to respond to and secrete interferon-γ [38]. We did not find that IL-12 secretion level was predictive of disease progression. However, IL-12 could promote resistance to HIV infection [39], and a previous study has showed that plasma IL-12 levels during acute HIV-1 infection predicted HIV disease progression [40]. Further studies are needed to clarify the impact of IL-12 secreted by CD1c + mDCs on disease development.

Although our results did not reveal whether TP and RP patients would benefit from early ART, they did suggest that a low CD1c + mDC count (<10 cells/μL) provided important prognostic information which could be used to guide therapy. Since patients with a low CD1c + mDC count are more at risk for rapid disease progression without therapy, such patients may be more likely to benefit from early treatment.

## Conclusions

In conclusion, CD1c + mDC counts and IL-12 secretion were positively associated with CD4+ T cell counts, and CD1c + mDC counts were inversely associated with viral loads. CD1c + mDC counts, CD86 expression, and IL-12 secretion level in RPs were lower than those in TPs. Low CD1c + mDC counts were correlated with a high risk of rapid disease progression during EHI, suggesting a potentially important prognostic marker and a promising target for intervention.
